# Challenges in sample preparation and structure determination of amyloids by cryo-EM

**DOI:** 10.1016/j.jbc.2021.100938

**Published:** 2021-07-03

**Authors:** Mara Zielinski, Christine Röder, Gunnar F. Schröder

**Affiliations:** 1 Institute of Biological Information Processing, Structural Biochemistry (IBI-7) and JuStruct, Jülich Center for Structural Biology, Forschungszentrum Jülich, Jülich, Germany; 2 Institut für Physikalische Biologie, Heinrich-Heine-Universität Düsseldorf, Düsseldorf, Germany; 3 Physics Department, Heinrich-Heine-Universität Düsseldorf, Düsseldorf, Germany

**Keywords:** cryo-EM, amyloid fibrils, structure determination, helical processing, EM software, Aβ, amyloid β, AD, Alzheimer's disease, CHEP, Clustering of Helical Polymers, CNNs, convolutional neural networks, cryo-ET, cryo-electron tomography, T2D, type II diabetes, YOLO, You Only Look Once

## Abstract

Amyloids share a common architecture but play disparate biological roles in processes ranging from bacterial defense mechanisms to protein misfolding diseases. Their structures are highly polymorphic, which makes them difficult to study by X-ray diffraction or NMR spectroscopy. Our understanding of amyloid structures is due in large part to recent advances in the field of cryo-EM, which allows for determining the polymorphs separately. In this review, we highlight the main stepping stones leading to the substantial number of high-resolution amyloid fibril structures known today as well as recent developments regarding automation and software in cryo-EM. We discuss that sample preparation should move closer to physiological conditions to understand how amyloid aggregation and disease are linked. We further highlight new approaches to address heterogeneity and polymorphism of amyloid fibrils in EM image processing and give an outlook to the upcoming challenges in researching the structural biology of amyloids.

### History of amyloid research

For a long time, the sole imperative in protein research was the Anfinsen dogma, which states that the fold of a protein is dictated by its primary sequence (1). Today, we suspect that all proteins may adopt another generic fold that is independent from their primary sequence—the amyloid fold (2, 3) (Fig. 1). The amyloid fold is characterized by the aggregation of proteins into stacks of β-sheets resulting in fibrils that exhibit a so-called cross-β pattern (2, 3, 4, 5). In the 19th century, the term amyloid was coined by Rudolf Virchow, by whom it was selected in reference to the Greek word for starch that Virchow considered the main building block of amyloid (6, 7, 8). A couple of years later, amyloid deposits were realized to be proteinaceous (6, 9). Until now, the findings that amyloids exhibit enhanced birefringence through Congo Red staining (6, 10, 11) and that they share the common cross-β pattern are considered the main characteristics of the amyloid fold. Currently, more than 50 amyloids (2, 12, 13) are linked to protein misfolding diseases such as Alzheimer’s disease (AD) (14), Parkinson’s disease (15, 16), and type II diabetes (T2D) (17), which have therefore been combined under the term amyloidoses (18). The proteins involved in these diseases often are intrinsically disordered proteins such as amyloid β (Aβ) (from AD) or amylin (from T2D), or at least contain a considerable fraction of disordered regions. Moreover, there are non–disease-related amyloidogenic proteins (19) such as the src-homology domain 3 that is known to form amyloids only *in vitro* (20). In addition, it has been observed that metabolites such as phenylalanine assemble into disease-causing amyloid-like aggregates (21). Functional amyloids that are mostly found in fungi or bacteria, where they often work as a defense mechanism against other species, have been identified, too (22, 23, 24, 25). Considering the ubiquity of amyloids, it is of great interest to understand why such a variety of proteins is able to adopt the fibrillar fold, either functional or pathogenic. Although amyloid structures share a common overall architecture, the mechanism of protein aggregation from an intrinsically disordered protein or globular fold into amyloid is not yet understood. While some amyloidogenic proteins adopt the amyloid fold only when exposed to extreme conditions such as very low pH, increased temperature, and shaking (20, 26, 27, 28), others seem to undergo proteolytic truncations (29, 30, 31, 32) before aggregation, and then again, there is a variety of proteins where a coincidental increase in local concentration seems to boost the misfolding reaction (33). Figure 1 visualizes different amyloid species and their putative misfolding pathways leading to either fibrils (on-pathway) or other aggregates (off-pathway). Following the on-pathway, disordered monomers aggregate into fibrillar oligomers that act as the precursor for maturation into fibrils (Fig. 1, lower part). These mature fibrils might then deposit together with other cellular components into insoluble plaques, which we can observe in diseased patients (34, 35). The off-pathway (Fig. 1, upper part), on the other hand, is considered to favor formation of amyloid oligomers that might aggregate into curvilinear fibrils (36, 37, 38). The biological relevance of these kind of aggregates remains elusive. However, in recent years, evidence accumulated that amyloid oligomers, especially those following the off-pathway, instead of amyloid fibrils represent the pathogenic species in amyloidoses (39, 40, 41, 42). However, the role of on- and off-pathway oligomers and amyloid fibrils in the aggregation mechanism and in pathology is not yet explained sufficiently (43, 44, 45). Accordingly, the understanding of amyloid fibril formation and their structures still is of high importance, for example, to increase our knowledge about amyloidoses as well as exploiting potential applications for functional amyloids.

**Figure 1:**
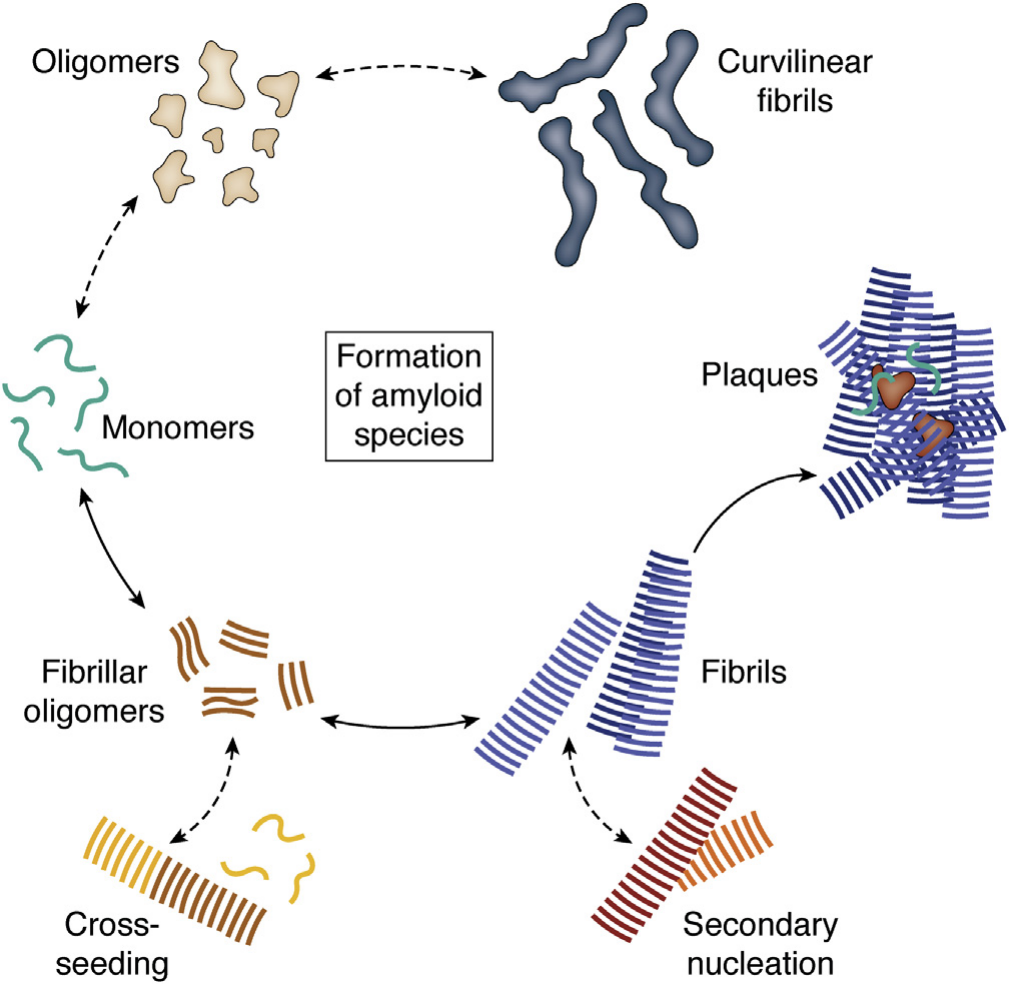
**Formation of structurally different amyloid species.** Starting from monomeric proteins (*green*), monomers can follow the on-pathway and aggregate into fibrillar oligomers (*brown*). Fibrillar oligomers will over time grow into mature fibrils (*blue*) that might, together with other species and molecules, deposit as plaques. Mature fibrils can undergo secondary nucleation during which monomers attach to a mature fibril and form a new fibril. In addition, fibrillar oligomers could seed formation of fibrils with monomers of a different protein (*yellow*), referred to as cross-seeding. Monomers might also follow the off-pathway and fold into oligomers (*beige*) and further into curvilinear fibrils (*dark blue*).

Notably, amyloid fibrils have many faces in that under the same conditions, the very same protein might aggregate into structurally different fibrils. This phenomenon is known as polymorphism. Polymorphism in amyloid fibrils can be found on at least two structural levels (Fig. 2*B*). First, protofilament polymorphism, which is mainly described on the secondary structure level of amyloids, namely their β-sheet conformation. Protofilament polymorphism can be subdivided into packing polymorphism (one monomer or protofilament exhibits different β-sheet bending) or segmental polymorphism (different sequence segments of the same peptide are part of the cross-β structure) (46, 47) (Fig. 2*C*). The second level of polymorphism might be described as ultrastructural polymorphism in which protofilaments assemble through different and/or multiple interfaces (Fig. 2*D*). Ultrastructural polymorphism is mainly based on the intermolecular interactions of mature fibrils or protofilaments (48). It can also encompass the defining parameters of amyloid fibrils such as rise, twist, width, and cross-over distance (Fig. 2*A*). While it was assumed that monomer structure can change within one fibril, it was recently described by Radamaker *et al.* (49) that these structural switches can occur multiple times within one fibril. These findings add even more complexity to the topic of amyloid fibril polymorphism.

**Figure 2:**
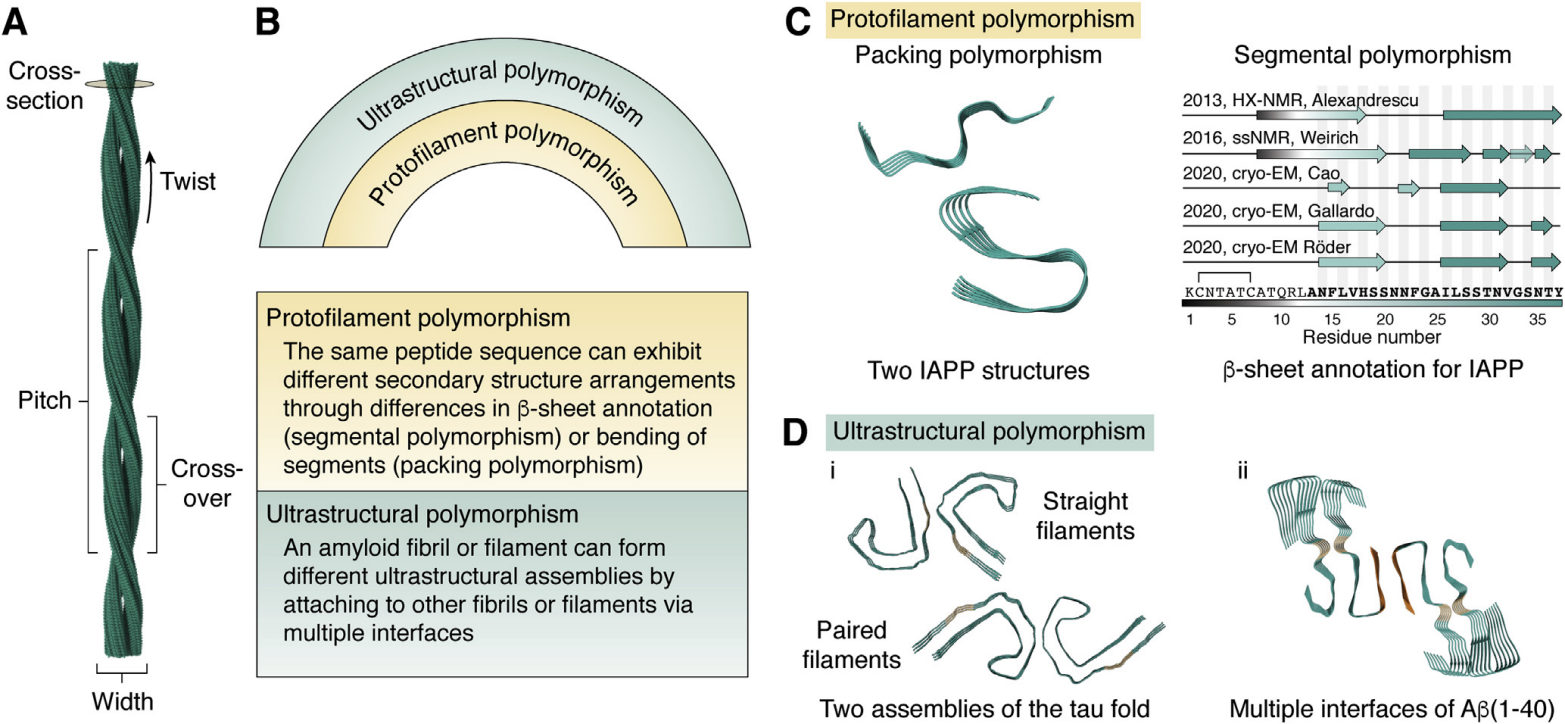
**Polymorphism in amyloids.***A*, an amyloid fibril is characterized by the helical parameters twist and rise as well as characteristic measures such as the pitch, crossover, and width. The cross-section usually is shown to display molecular arrangement and polymorphism. *B*, two levels of polymorphism can be distinguished in amyloid fibrils that can occur in different compositions: protofilament polymorphism, which can be subdivided into segmental or packing polymorphism, and ultrastructural polymorphism. *C*, examples for protofilament polymorphism. Packing polymorphism is displayed by the example of islet amyloid polypeptide structures whose segments bend differently depending on pH. Segmental polymorphism has also been revealed for islet amyloid polypeptide as the assignment of β-sheets is different (87, 88, 89, 195, 196). *D*, examples of ultrastructural polymorphism. i, the general tau fold can exhibit different interfaces resulting in straight (*left*) or paired (*right*) helical filaments. ii, Aβ(1–40) reveals multiple interfaces (*beige* and *orange*) that result in double fibrils (47). Structures shown: 6Y1A (87), 6VW2 (88) (*C*); 6HRF, 6HRE (197) (*D*(i)); 6SHS (92) (*D*(ii)).

### Structure determination of amyloid fibrils

Although there have been many insights regarding both the diversity of amyloid fibrils and their commonly shared architecture, structural details on the atomic level remained hidden for a long time. The first high-resolution atomic structures of amyloid fibrils were published in the 2000s by means of solid-state NMR spectroscopy assisted by X-ray fiber diffraction and transmission electron microscopy (4, 50, 51). NMR and X-ray diffraction are established tools for protein structure determination, but when it comes to amyloid structures, they both show drawbacks. NMR requires high sample load and isotopic enrichment that often is expensive. Owing to the repetitive nature of amyloid fibrils, NMR signals are usually strong. But because amyloid fibril samples tend to be polymorphic, with NMR, it is hard to differentiate between less-populated polymorph species.

Amyloid crystals consist of small and untwisted fragments from amyloidogenic proteins (52) because crystallization of mature amyloid fibrils (Fig. 2*A*) is impossible. Hence, together with the start of the “resolution revolution” in cryo-EM, a new era began for our understanding of the structural features of amyloidogenic proteins in general and amyloid fibrils in particular. In 2017, the first high-resolution fibril structures of two of the main disease-related amyloids, tau and Aβ1-42, were solved by Fitzpatrick *et al.* (53) and Gremer *et al.* (54) using cryo-EM. With resolutions of 3.4, 3.5 Å (tau) and 4.0 Å (Aβ1-42), atomic model building with cryo-EM data became possible for the first time. Since then, over 70 amyloid cryo-EM structures have been published, most of which reach resolutions of 3 to 4 Å. This resolution allows for distinction of different folds within one sample and often also for atomic model building. However, higher resolutions will be important for identifying posttranslational modifications or cofactors and for a better understanding of, for example, secondary nucleation mechanisms. The atomic resolution barrier, for which the threshold is considered to be 1.2 Å (55), has already been broken in 2020 for large globular protein complexes by single-particle cryo-EM (56, 57). In the field of amyloid research, the highest resolution achieved so far is 2.3 Å (58). However, we are optimistic that the rapid progress in the field of sample preparation and structure determination of amyloid fibrils by cryo-EM will allow us to break the atomic resolution barrier in the future.

### Development of cryo-EM

Cryo-EM is based on transmission electron microscopy, which has been developed in the late 1960s. Biological samples first came into play with the development of negative stain EM (59) that led to the first 3D structure of the extended tail of the T4 bacteriophage (60, 61). To no surprise, this sample exhibits helical symmetry because helical samples have two intrinsic advantages over globular proteins: first, all necessary information is sufficiently provided by one single image, and second, the repeating asymmetrical units in a helical filament show fixed relative orientations. Relative orientations can be deduced from the helical symmetry parameters twist and rise (Fig. 2*A*) (62). Thus, the level of noise can be significantly decreased by averaging over many asymmetrical units (63, 64). For amyloid fibrils, the cross-β arrangement highly facilitates the determination of helical symmetry (65). However, the alignment along the helical axis is almost exclusively based on the 4.7 Å (meridian) signal caused by the cross-β pattern (stacking of several β-sheets along the fibril axis) and a 10 Å (equatorial) signal due to the horizontal interstrand distances within a molecule (66). The predominance of the 4.7 Å signal and the lack of larger structural features may complicate the high-resolution structure determination of amyloid fibrils (65).

Cryo-EM enables the imaging of radiation-sensitive samples under cryogenic conditions, which reduce radiation damage resulting from the interaction of the sample with high-energy electrons. The discovery of the advantages of samples frozen in vitreous ice (67, 68) together with the development of practical applications (69) drastically advanced the field of cryo-EM in general. But still, for years, cryo-EM has been mockingly referred to as “blobology” because the obtained resolutions have been rather low and the process itself was slow. In short, the development of direct electron detectors (70, 71, 72) as a replacement for photographic film or charged coupled device cameras led to a big leap in the field. The advancement of cryo-EM into a powerful and widely used tool in structural biology has also substantially been shaped by progress in computation. Because of their inherent advantages described above, helical structures were among the first to be described by cryo-EM (73, 74, 75). However, the computational development that made cryo-EM broadly applicable was the single-particle reconstruction, which is based on the groundwork of Joachim Frank (76). Together with a technique referred to as (Box 1) “projection matching” developed by Frank and Penczek (77) and the reconstitution method by Marin van Heel (78), which made sample tilting redundant, these techniques built the basis for today’s software packages (https://www.emdataresource.org/emsoftware.html). According to Electron Microscopy Data Bank statistics, RELION (79) that implements a (Box 1) Bayesian approach to structure determination is the most used software contributing to nearly 50% of all released maps (https://www.ebi.ac.uk/pdbe/emdb/statistics_software.html/). In the field of helical reconstruction, major breakthroughs were the application of Fourier–Bessel principles to compute 3D models (60) and the development of the (Box 1) iterative helical real-space reconstruction method (80), which forms the basis of all popular reconstruction programs that are in use today.

Box 1 **Glossary**

**Template matching**

Template matching is an image-processing technique that can be used to find images similar to a template by maximizing the cross-correlation between the template and all images.

**Convolutional neural network**

Convolutional neural networks (CNNs) are a category of artificial neural networks mostly used in the field of image analysis; however, they can also be used for other data analysis tasks as well as classification problems. A CNN, which is inspired by biological processes, is specialized to pick or detect and interpret patterns.

**Sliding-window approach**

The sliding-window approach is often used in the implementation of a CNN. Here, the image is processed by sliding a rectangular window over the whole image to pass different portions of it through a CNN.

**Projection matching**

Projection images of an initial reference model are compared with the experimental EM images of the protein, and orientations are assigned based on the highest cross-correlation between projection images and experimental image.

**Fourier–Bessel Inversion**

The analysis of the helical symmetry as well as the calculation of a 3D reconstruction are conducted in Fourier space. The approach is based on Klug’s theory of diffraction patterns of helical structures (192) and Cochran’s interpretation of helical filaments as curled-up 2D lattices in Fourier space (193).

**Single-particle approach to helical reconstruction**

The single-particle approach to helical reconstruction provides an alternative to Fourier–Bessel inversion. The image of a helical protein is divided into equally sized overlapping segments that are treated as individual images, analogous to single particles. Relative orientations of segments are determined by projection matching.

**Iterative helical real-space reconstruction algorithm**

The algorithm (80) uses the single-particle approach to helical reconstruction and iteratively carries out the steps of projection matching. In-between iterations, the helical symmetry is imposed onto the reconstructed volume that is used as a reference in the next iteration. This way, the density map is optimized in every iteration.

**Bayesian approach to structure determination**

An image does not get assigned a single orientation but an entire distribution of weighted orientations (the likelihood function). In the Bayesian formalism, this likelihood can be combined with prior knowledge about parameters (*e.g.*, a range of orientations that we think is relevant). This procedure leads to a more robust optimization and allows for a more formal treatment of prior knowledge and errors (64, 194).

In this review, we will focus on the combined growth of the cryo-EM and the amyloid field, especially in regard to computational or technical challenges and advances on amyloid fibril reconstruction (Fig. 3). We aim at clarifying the challenges we see in sample preparation of amyloid fibrils for cryo-EM and what (computational) difficulties are faced during reconstruction. In addition, we are taking a closer look at recent technical advances in both sample preparation as well as 3D reconstruction. Finally, we conclude with future developments that might help moving the field of amyloid structure research forward. This review aims to make researchers in the field of amyloid aggregation as well as cryo-EM scientists aware of the intricacies and challenges in cryo-EM structure determination of amyloids and encourage method developers to help with new tools. For further information on amyloids in general, we would like to refer to recent excellent reviews by Iadanza *et al.* (13) and Ke *et al.* (52).

**Figure 3:**
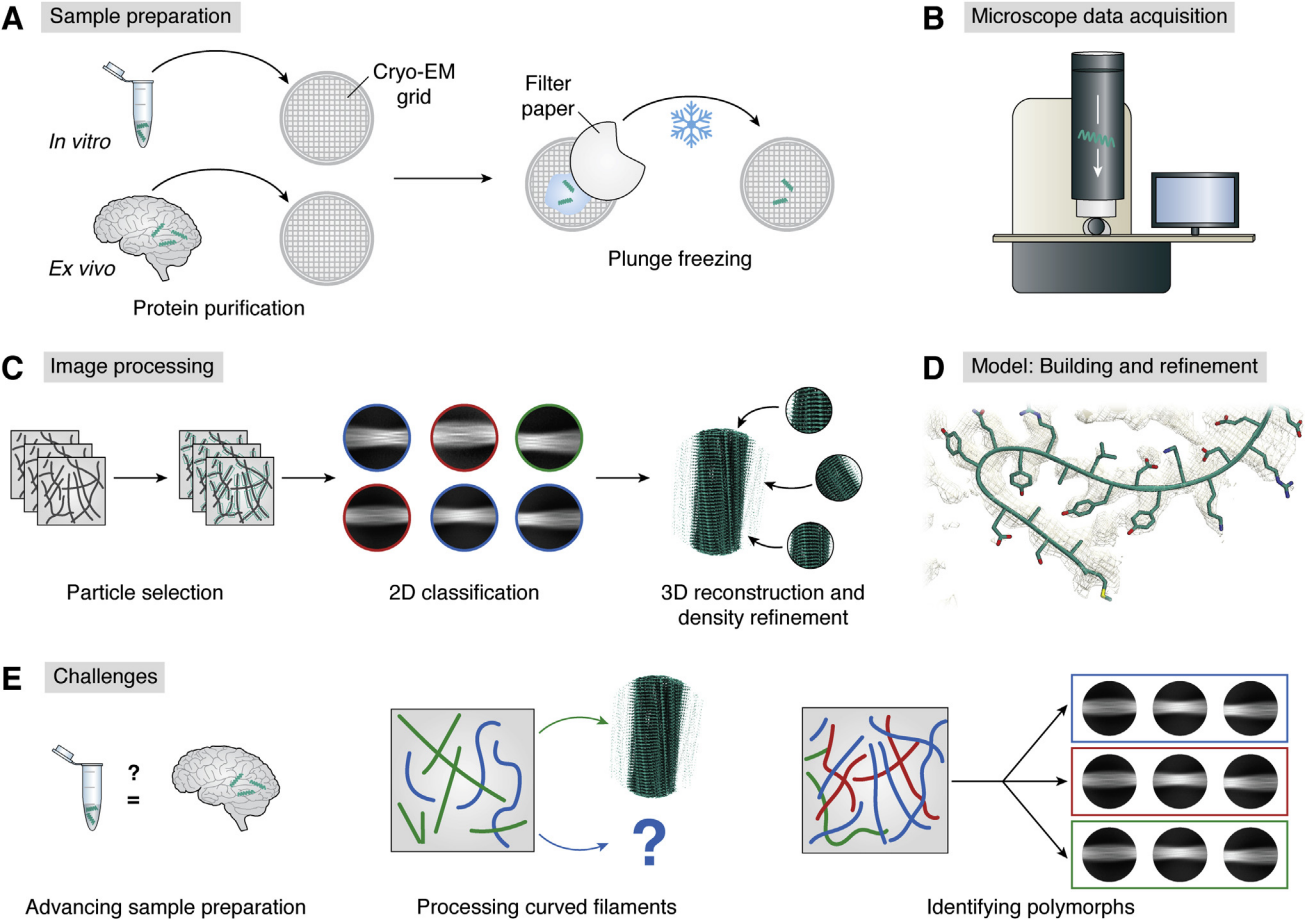
**Cryo-EM workflow.***A*, followed by protein purification, the purified sample in the solution is applied onto the cryo-EM grid (sample carrier), and excess buffer is blotted off using filter paper (*white shape*). In a subsequent step, the sample is plunge-frozen in a thin layer of vitreous ice. *B*, a dataset is collected after screening and assessment of the vitrified cryo-EM grids at a cryo-electron microscope equipped with a direct electron detector. *C*, image processing of the acquired data involves several steps, major ones being particle selection, 2D classification, as well as 3D reconstruction and refinement of the density map. Amyloid fibrils are selected from the preprocessed (*e.g.*, motion-corrected and contrast transfer function-estimated) micrographs. In the following step of particle extraction, selected fibrils are segmented into so-called particle images. Subsequently, in 2D classification, particles that show similar views of the protein are grouped together, thereby improving the signal-to-noise ratio. Two-dimensional classes that show invalid or contaminated data are removed from the dataset. In a next step, particles from 2D classes are used to calculate 3D density maps. *D*, as soon as a high-resolution reconstruction is obtained, an atomic model can be built and refined. *E*, challenges in amyloid structure determination: advanced sample preparation approaches (*left*), processing curved filaments (*center*), and identification of polymorphs as part of the automated particle selection process (*right*).

## Main text

### Sample preparation for amyloids moves closer to *in vivo* conditions

For cryo-EM, the sample protein does not have to be isotope-labeled (NMR) or crystallized and treated with special crystallization screening solutions (X-Ray) to prepare a usable sample. However, also for cryo-EM, the sample preparation needs to be optimized to find conditions that not only lead to thin ice and a favorable particle distribution but also result in stable and homogeneous proteins or protein complexes. This is also true for amyloid fibrils, for which a major challenge during sample preparation is to ensure the formation of individual, well separated but sufficiently concentrated single fibrils.

In general, sufficient protein stability can be very difficult to achieve for globular proteins and membrane proteins in particular. In contrast, the mature amyloid fibril state is considered very stable (81, 82, 83). But the understanding of the fibril formation process is elusive and buffer conditions including but not limited to pH, salt concentration, temperature, and pressure are believed to have a strong impact on aggregation pathways of amyloids into either rigid, mature fibrils or curvilinear (proto-)fibrils and oligomers (84) (Fig. 1). In addition, small differences in the buffer system or introduction of physical forces (*e.g.*, beads) might change aggregation kinetics (27, 85, 86) or induce the formation of ultrastructrual polymorphs (87, 88, 89).

However, which polymorphs actually appear *in vivo* and are disease-relevant remains an important question (Fig. 3*E* left). Consequently, one approach is to work with *ex vivo* samples that are extracted from tissue (90, 91) (Fig. 3*A*). But purification from cells or organs is challenging especially because of amyloid properties such as the lack of known specific binding partners that could be used for purification. Amyloid species tend to clump, which makes them usually very inhomogeneous in size and hard to separate from other cell components. Moreover, amyloid fibrils can be found as single fibrils of different lengths or as fibril bundles of different mass, for example, Aβ plaques in AD. *In vivo*, there often are several species of one amyloidogenic precursor protein (*e.g.*, Aβ1-42, Aβ1-40, Aβ2-42, etc., in AD (92)) in an inhomogeneous, polymorphic mixture. While polymorphs can already be quite difficult to be distinguished on micrographs, differences in the monomer length might be even more difficult to distinguish if they do not form very different polymorphs. In addition, amyloid formation *in vivo* might result in structural bias because the distinct, probably patient-dependent, environment could lead to preferred formation of one polymorph over another. Structural bias could be introduced as well through harsh extraction conditions that could potentially alter at least the solvent-exposed parts of a fibril. In 2016, Annamalai *et al.* (93) described ultrastructural features of patient-derived fibrils. Although the number of high-resolution *ex vivo* structures has been continuously growing since (53, 58, 92, 94, 95, 96, 97, 98, 99), it remains challenging to use tissue-extracted amyloid fibril material for use in cryo-EM. As a workaround, patient-extracted tissues have been used to seed fibril growth for subsequent structural studies (90, 100, 101). Here, monomers of the amyloidogenic proteins are added in excess to homogenized tissue to amplify the existing *ex vivo* fibrils. This procedure is based on the assumption that the existing aggregates in the tissue will seed the growth of aggregates, fibrils in particular, of the same polymorph (101, 102, 103). The observed amplified sample is thus not directly taken from human tissue but is a more indirect representation of *in vivo* structures than *ex vivo* structures.

Interestingly, none of the few known *ex vivo* fibril structures have been observed either *in vitro* or in amplified patient-derived samples, yet (99, 104). Nonetheless, *in vitro* structures have broadened our understanding of amyloid architecture in general and revealed common structural features such as the amyloid key, which has been observed *in vitro* and *in vivo* and they furthermore help interpret aggregation kinetics (105, 106). Moreover, *in vitro* structures provided valuable insights regarding the improvement of fibril preparation and fibril reconstruction. Although the example of an amyloid key shows general overarching patterns, the transferability of *in vitro* data to the mechanisms and structures forming in organisms remains unclear. Hence, one future goal is to enable the *in vitro* production of disease-relevant amyloid aggregates (not necessarily restricted to fibrils), which are needed to study the molecular disease mechanism and facilitate drug development, by reproducing *in vivo* findings in the test tube. Notably, several of the *ex vivo* structures show incorporation of small molecules, which very likely are important for stabilizing the observed polymorph. Reproducing the *ex vivo* polymorphs would therefore require to identify these cosolutes and add them to *in vitro* fibrillization assays. Without better knowledge of the *in vivo* fibrillization conditions, it will therefore be very difficult to form the polymorphs that are, for example, involved in disease progression, in *in vitro* experiments.

The same holds true for amyloids consisting of more than one protein. Until now, amyloid fibril structures have been exclusively determined from a single sort of protein, although coaggregation (107) of different amyloidogenic proteins has been described.

### Amyloid fibrils could benefit from novel vitrification tools

Plunge-freezing (Fig. 3*A*) was first established as a manual technique (68, 69). Later, automated and commercial plunging tools were developed (*e.g.*, the Vitrobot (108)), with the goal to achieve reproducibly good ice thickness, less contaminations, and homogeneous vitrification of the sample. Newer developments of grid preparation devices mostly aim to replace the blotting step, which is hard to control precisely, leads to nonuniform ice thickness, and adds significantly to the lack of proper reproducibility (109).

For amyloid fibrils, we often observe that sample concentrations on the grid vary drastically between replicates of the same origin concentration, which might be caused by blotting. Blotting could affect helical structures more than single particles because an attachment of one part of a filament to the blotting paper could potentially remove a whole network of filaments from the grid. Furthermore, the large hydrodynamic forces exert considerable stress that could affect filaments and fibrils. And again, this stress can be expected to affect filaments much more than individual globular protein complexes because filaments extend over longer distances. For example, the filament variability (such as filament twist) and also the subunit conformation of F-actin have been shown to be susceptible to forces on the filament (110, 111, 112). How much these hydrodynamic forces affect the structure of amyloid fibrils remains to be studied.

The Chameleon system (introduced under the name Spotiton (113, 114, 115, 116)) transfers a drop of picolitre volume onto the EM grid automatically. The use of such a small sample volume makes the step of blotting unnecessary, which could be of advantage for sample preparation of amyloid fibrils as explained above. Similarly, the cryoWriter (117, 118, 119) only needs some nanolitres of sample and uses a microcapillary to apply the sample to the grid while the grid is moving (hence “writer”). An IR laser controls for the perfect film thickness before plunge-freezing automatically (120). During the process, the cryoWriter does not create aerosols during the application step, which is important for amyloid samples, where there are hints toward the transmissibility of disease *via* protein samples. Experiments with mice showed that Aβ pathology can be transferred from human tissue samples through intracerebral inoculation of pathogenic amyloid (121). The study was based on former findings of initial evidence for Aβ transmission *via* neurosurgery (122, 123). On the other hand, retrospective studies show no increased risk for AD from blood transfusions in-between humans (124, 125). However, because the infectiousness of amyloid samples is unclear, precautions should be taken when handling these samples.

Comparable with the process of 3D printing, the VitroJet (126, 127) “prints” the sample solution onto the EM grid in a layer thin enough to make the blotting step obsolete. Instead of plunge-freezing, the cryogen is sprayed onto the grid directly, which shall ensure faster and more homogenous vitrification that would be advantageous for all kinds of samples.

An affordable DIY grid preparation device has been introduced by Rubinstein *et al.* (128) with the Shakeit-off. It is based on a simple USB ultrasonic humidifier that sprinkles the sample across a self-wicking nanogrid (129, 130) before subsequent vitrification. Owing to the undirected spreading of the sample and the production of aerosols, the system would need further adaptation to shield the user from the spread of hazardous samples.

Some of the grid preparation devices mentioned above and their ability to improve the distribution within the air–water interface have been analyzed by Klebl *et al.* (131). Other techniques (108, 115, 118, 127, 128) and sprayer designs (132) proposed in the last years are yet not widely used and thus will have to prove their practicality on amyloid samples in the future. It would also be an additional benefit if the next generation of plunge-freezing devices were able to orientate or align filaments on the grid because identical orientations of fibrils would facilitate particle picking and subsequent 3D reconstruction.

### Automated (pre-)processing is on the rise for filaments

Because of the variety of cryo-EM software and complexity of settings, structure determination with cryo-EM data is highly dependent on individual skills and expert knowledge (133). Automated (pre-)processing, including all steps between data acquisition and 3D reconstruction (Fig. 3, *B* and *C*), might help making the technique more widely accessible (134). Commonly used software packages that include automated preprocessing steps are, for example, cryoSPARC (135), SPHIRE (136), and RELION 3.1 (79).

Amyloid fibril reconstructions are challenging and usually require extensive manual work. One reason for this is that the 4.7 Å-spaced cross-β pattern is very dominant and creates many false local optima during the simultaneous determination of particle orientation and helical symmetry parameters. For example, the directions of fibrils, described by the psi angle, usually converge rather slowly. Furthermore, because the fibril twist is energetically not strongly restrained, variations in helical symmetry can occur between different fibrils and even along a single fibril. In addition, the data often show different polymorphs with only small structural differences that are difficult to sort out (100).

Particle selection from electron micrographs lays the groundwork for a high-resolution reconstruction. Manual picking of particles is still considered the most accurate way of particle selection although it is very time consuming, especially for large datasets. There are various software tools that implement an algorithm for (semi-) automated particle selection mostly based on (Box 1) template matching (137, 138, 139, 140) or (Box 1) convolutional neural networks (CNNs) (141, 142, 143, 144, 145). Typically, in a first step, a small subset of the entire dataset needs to be picked manually either to train the CNN or to generate template images. Subsequently, automated particle picking of the entire dataset can be performed. For data with grid-like topology, CNN-based methods are lately the best choice for pattern recognition and object detection. While most tools that implement CNNs are based on the so-called (Box 1) sliding-window approach, crYOLO (143) implements the deep-learning object detection system You Only Look Once (YOLO) (146). In contrast to the computationally expensive sliding-window approach, the YOLO algorithm speeds up calculations to six micrographs per second on one graphics processing unit. In the future, this might be supported by the automated particle-diameter estimation by Li *et al.* (134), a single-particle tool that performs automatic particle-diameter estimation and supersedes the training need for crYOLO by determination of the correct box size for picking through a trial-and-error approach. Automatic picking of amyloid fibrils remains challenging because of the need of avoiding the selection of crossings and overlaps of fibrils as well as the start- and end-point of a fibril not necessarily being visible on the image frame. Recently, filament selection has been implemented in crYOLO (147), and it may open the door for the transition from time-consuming manual particle selection to automated selection also in the field of amyloid fibril structure determination.

Recently, Thurber *et al.* (148) presented FibrilFinder, an approach to fully automated particle selection without requiring prepicking of the dataset. Although the algorithm avoids picking fibrils on carbon, it cannot detect intersections of fibrils. Subsequent use of their program FibrilFixer, which can be applied after particle extraction, discards particle images that show fibril intersections. The latter one could also be a helpful stand-alone tool after manual particle selection. Here, picking intersections is usually avoided, and as a result, one fibril is split into several smaller fibril segments, causing a loss of information about angular correlations. Recovering this information could facilitate image alignment. Both FibrilFinder and FibrilFixer are directly compatible with RELION-3.1, making them easy to integrate into the image processing workflow.

MicHelixTrace (149), which is available in the SPRING (150) software suite, locates helical filaments on micrographs by using a 2D class average as the reference image. The detection of filament positions on the micrographs is based on a cross-correlation map that is generated by the determination of the rotation and translation of small, straight fibril segments relative to the reference image. Moreover, the program determines the persistence length of each filament, which may be used to characterize mechanical properties and assess the potential for the reconstruction to reach high resolution.

In the last few years, major steps in the field of automated (pre-)processing have improved the workflow. For globular proteins, automated selection often is not only faster but possibly even more precise than manual particle selection. Similarly, automation tools already assist in the preprocessing of amyloid reconstruction; however, it is not yet expedient to perform entirely automated processing. At least at the current stage, where, for example, automated selection of helical filaments still suffers from inaccuracies such as insufficient identification of different polymorphs, manual inspection of the results is indispensable.

### New tools facilitate initial model generation

As mentioned above, the search for orientation and helical symmetry parameters suffers from many false local optima (151). Determination of a robust initial estimate of the 3D structure greatly facilitates subsequent density refinement and image classification (Fig. 3*C*). RELION 3.1 offers a novel approach to calculate an initial 3D model *de novo* (65). The model can be calculated either from one 2D reference-free class average that shows an entire crossover or from several class averages that, given some additional information, can be combined to calculate an initial 3D density. Another method to produce an initial model by combining several 2D class averages was presented by Ghosh *et al.* (101) in their structural study of an Aβ polymorph from AD brain tissue. Moreover, introducing additional orientation parameter regularization avoids local optima. Ghosh *et al.* (101) used a modified version of the software package RELION 3.0 beta (79) for 3D reconstruction.With their modifications, angular restraints between neighboring segments from the same fibril were introduced to make the image alignment during 3D reconstruction in RELION 3.0 more robust and consistent. In addition, a method to increase the precision of the 2D alignment before moving to the step of 3D reconstruction was introduced.

### Curvature of amyloid fibrils remains a challenge in 3D reconstruction

Amyloid fibrils tend to be flexible and therefore rarely appear as straight filaments on electron micrographs. Especially, long fibrils tend to be entangled in a fibril network and have a higher chance of interacting with surfaces. They are more susceptible to hydrodynamic forces and therefore cannot as easily relax into a straight conformation as short fibrils. However, for classical helical reconstruction, it is necessary to use the straight parts of a fibril because the applied helical symmetry ignores bending, which can limit the resolution (Fig. 3*E* center). The resolution could also be further improved when releasing the symmetry altogether during the refinement and treat segments of the fibrils as single, independent particles (152). If the fibrils are strongly curved, the total amount of usable data could be limited because strongly curved regions of the fibrils need to be excluded from processing. To include bent filaments, Ohashi *et al.* (153) introduced a novel soft-body model for 3D reconstruction instead of the classical rigid body. Through introduction of hidden parameters, which define the curvature of a fibril, it allows for optimization of 3D reconstruction based on Bayesian inference.

### Amyloid structure determination requires polymorph identification

The morphological composition of an amyloid fibril sample is a valuable information which shows the structural diversity of an amyloidogenic protein and its sensitivity to environmental conditions. As of now, the image processing (Fig. 3*C*) of different polymorphs is performed separately comparable with the processing of different protein conformations. As a consequence, the application of tools for automatic particle selection on highly heterogeneous datasets of amyloid fibrils is severely limited because even the most accurate algorithms still fail to distinguish between different polymorphs (Fig. 3*E* right). Thus, fibril characteristics such as the fibril diameter and the cross-over distance (Fig. 2*A*) are usually determined manually, for example, from 2D classes (154) or micrographs. Interestingly, two different monomer folds, which were observed within one fibril polymorph, could be separated in 3D classification for a sample from amyloid light-chain amyloidosis (49). However, the separation of different morphologies by hand is a laborious and error-prone task. But, manual polymorph selection can be facilitated by gold nanoparticles that have been shown to attach to the surface of a large variety of amyloid fibrils and hence can be used for fibril characterization (155). With their small size and high electron density, these gold nanoparticles make helical characteristics such as pitch and crossovers well visible in electron micrographs and hence allow for easy differentiation between polymorphs (Fig. 2).

An alternative and less-laborious approach to manual selection is the automated detection of cross-overs in images of amyloid fibrils, which can be achieved by applying conventional computer vision techniques combined with machine learning approaches (156). This method enables the statistical analysis of the sample and thus gives insights into its morphological composition. However, it is yet not possible to incorporate automatic crossover detection into the processing workflow in a way that datasets can be separated into subsets of different polymorphs automatically. The latter can be achieved by applying Clustering of Helical Polymers (CHEP) (157, 158), an algorithm which clusters polymorphs into homogeneous groups, each representing one polymorph through its conformation, composition, and/or helical symmetry. The polymorph separation is achieved by the combination of 2D classification results and information about the association of a particle image to the originate fibril. Hence, automated particle selection for amyloid fibrils may be facilitated by CHEP, separating the original dataset into homogeneous subdatasets. In addition, the method can likewise be used for statistical analysis of the morphological composition of the sample. Moreover, by applying CHEP to amyloid datasets, the final resolution might increase because for low-contrast micrographs, the manual separation of one dataset into homogeneous subsets is difficult. Here, errors are likely to be introduced because of incorrect picking, which inevitably causes a decrease in the final resolution of the reconstruction. Hence, to be able to compute a high-resolution reconstruction, it is of major importance to detect different polymorphs with high precision. Especially, for small structural differences between different polymoprhs, it is therefore desirable to develop tools that can either assist in manual identification or enable the use of automatic selection tools, which until now lack sensitivity to select different polymorphs separately.

## Outlook

### Time-resolved cryo-EM for understanding amyloid formation

After the ground-breaking work by Nigel Unwin (159) that introduced time-resolved cryo-EM, recent developments further pushed time-resolution to the order of milliseconds by mixing reactants with microfluidics devices (160, 161). For vitrification, the chameleon system has recently been modified to prepare samples for time-resolved cryo-EM (162). However, amyloid aggregation is a rather slow process and usually occurs on the time scale of minutes up to weeks and would therefore not even require sophisticated sample preparation methods. It is usually possible to simply prepare grids at many time points during the aggregation process, thereby creating snapshots of the structural evolution of different aggregates and polymorphs. Such a time-dependent analysis of amyloid formation has, however, not been extensively exploited so far. Observing the time evolution of the distribution of aggregating amyloids as, for example, a shift in the oligomer to fibril ratio over the course of minutes to days or appearance of different polymorphs with different kinetics and potential transient aggregates would be very insightful to understand the aggregation pathways and aggregation mechanisms and how different species are connected. Even negative-stain EM could yield valuable information on the structural landscape at low resolution. The main challenge is, however, the structural heterogeneity, which makes it difficult to identify specific polymorphs and to separate them during the image processing, in particular for polymorphs that are only weakly populated.

### Heterogeneous amyloid aggregates will be future cryo-EM targets

Since 2017, the number of high-resolution structures of amyloid fibrils has increased rapidly, while at the same time the amyloid field has made great progress in describing the formation of different amyloids. It is common agreement that amyloids form through several pathways that include monomeric self-assembly (163, 164, 165), secondary nucleation (166), and (cross-)seeding (167) (Fig. 1). While the amyloid structures we know to date are based on monomeric self-assembly or homonuclear seeding, other pathways are much more difficult to observe because of their comparably low occurrence. Although cryo-EM experiments could show secondary nucleation on an ultrastructural level (168), high-resolution details of this process are still lacking. First insights might be given by an interesting cryo-EM structure of amylin comprising two identical and one different protofilament that has recently been described and possibly is showing secondary nucleation (89). On the other hand, cryo-electron tomography (cryo-ET) in combination with cryo-focused ion beam milling can give much more insight into irregular and heterogeneous aggregates, especially in a cellular environment as, for example, described by Bäuerlein *et al.* (169) who visualized polyglutamine inclusions from Huntington’s disease in neurons. In addition, cryo-ET might also shed some light on amyloid fibrils that do not show any apparent twist (170). A missing twist could be an inherent property of a fibril but also an artifact from a close contact or attachment to the air–water interface (171). Structures of amyloid fibrils without twist (100, 106) lack helical symmetry and therefore cannot be solved using standard helical reconstruction techniques.

Interestingly, there have been indications that certain amyloidoses may trigger each other’s progression as, for example, for T2D and Parkinson’s disease (172, 173) or AD (174). Hence, the cross-seeding ability of the respective disease-associated proteins has been examined and confirmed (175, 176, 177). Structural data on this phenomenon would be of high impact and could potentially be observed using cryo-ET.

### Recent interest in structure determination of oligomeric amyloid species

In recent years, the toxic oligomer hypothesis (41, 178, 179, 180), which states that small, soluble oligomers are the most toxic amyloid species, has gained popularity. These soluble oligomers are therefore high up on the list of potential drug targets for several neurodegenerative diseases. There is therefore a strong interest in determining structures of amyloid oligomers for rational drug development to either prevent oligomer formation or disassemble them (181, 182), develop diagnostic tools based on oligomers (183), or evaluate the failing of previous drug candidates targeting oligomeric states (184, 185). It is of great interest to understand how monomers fold into oligomers and what the structural difference is between oligomers that are on-pathway toward fibril formation and those oligomers that do not further evolve into fibrils (36, 186, 187) (Fig. 1). However, because oligomers are structurally highly heterogeneous with a high variability in size and shape, it is challenging to determine their structure, but it might be possible to study them by cryo-EM. *In situ* structure determination by cryo-ET allows the analysis of aggregation processes of heterogenous samples and rare species in their cellular context by examining, for example, cellular sections. It is currently applied for amyloids (169, 188), and we assume it will play an important role in oligomer research in the future, if it improved in resolution. On the other hand, single-particle cryo-EM already delivers high-resolution structures, and *in vitro* oligomeric species such as the dimAβ construct, a toxic Aβ oligomer that forms homogeneously and reproducibly out of Aβ dimers (189), could work in favor of cryo-EM measurements. For visualization of small proteins, the usage of fragment antigen binding has been established (190) and applied for amyloid fibrils already (191). Likewise, fragment antigen binding could enhance the visibility of small oligomeric species in cryo-EM datasets by increasing the total complex size and introducing a reoccurring feature for alignment.

### Summary

The last years have shown that amyloid fibrils can be very well studied by cryo-EM even to high-resolution, which has provided exciting new insight into the architecture of amyloid fibrils. We have shown that cryo-EM of amyloid fibrils benefit from several recent technological advances. The apparent discrepancy of *ex vivo* and *in vitro* structures shows that we have not yet sufficiently understood how to mimick the physiological conditions for fibril formation *in vitro*, which remains an important next question to be answered. Finally, the structure of other aggregates such as toxic oligomers will hopefully soon complement our picture of amyloids.

## Conflict of interest

The authors declare that they have no conflicts of interest with the contents of this article.
